# *In vivo* Inhibition of the 3-Dehydroquinate Synthase by 7-Deoxysedoheptulose Depends on Promiscuous Uptake by Sugar Transporters in Cyanobacteria

**DOI:** 10.3389/fmicb.2021.692986

**Published:** 2021-06-23

**Authors:** Johanna Rapp, Berenike Wagner, Klaus Brilisauer, Karl Forchhammer

**Affiliations:** Interfaculty Institute of Microbiology and Infection Medicine, Organismic Interactions, Eberhard Karls Universität Tübingen, Tübingen, Germany

**Keywords:** 7-deoxysedoheptulose, allelopathy and allelochemicals, sugar uptake, 3-dehydroquinate synthase, shikimate pathway inhibitors, cyanobacteria, fructose ABC-transporter, glucose permease

## Abstract

7-Deoxysedoheptulose (7dSh) is a bioactive deoxy-sugar actively excreted by the unicellular cyanobacterium *Synechococcus elongatus* PCC 7942 (*S. elongatus*) but also *Streptomyces setonensis*. In our previous publications we have shown that in *S. elongatus*, 7dSh is exclusively synthesized by promiscuous enzyme activity from an inhibitory by-product of radical SAM enzymes, without a specific gene cluster being involved. Additionally, we showed that 7dSh inhibits the growth of cyanobacteria, but also the growth of plants and fungi, presumably by inhibiting the 3-dehydroquinate synthase (DHQS), the second enzyme of the shikimate pathway, as the substrate of this enzyme strongly accumulates in cells treated with 7dSh. In this study, by using purified DHQS of *Anabaena variabilis* ATCC 29413 (*A. variabilis*) we biochemically confirmed that 7dSh is a competitive inhibitor of this enzyme. By analyzing the effect of 7dSh on a subset of cyanobacteria from all the five subsections, we identified different species whose growth was inhibited by 7dSh. We also found that in some of the susceptible cyanobacteria import of 7dSh is mediated by structurally different and promiscuous transporters: 7dSh can be taken up by the fructose ABC-transporter in *A. variabilis* and via the glucose permease in *Synechocystis* sp. PCC 6803 (*Synechocystis* sp.). In both cases, an effective uptake and thereby intracellular enrichment of 7dSh was essential for the inhibitory activity. Importantly, spontaneous mutations in the sugar transporters of *A. variabilis* and *Synechocystis* sp. not only disabled growth of the two strains on fructose and glucose, respectively, but also almost abolished their sensitivity to 7dSh. Although we have clearly shown in these examples that the effective uptake plays an essential role in the inhibitory effect of 7dSh, questions remain about how 7dSh resistance works in other (cyano)bacteria. Also, the involvement of a putative ribokinase in 7dSh resistance in the producer strain *S. elongatus* remained to be further investigated. Overall, these data establish 7dSh as the first allelochemical targeting the shikimate pathway in other cyanobacteria and plants and suggest a role of 7dSh in niche competition.

## Introduction

Cyanobacteria colonize the highly diverse habitats of the entire illuminated biosphere. They are found in aquatic habitats–either in salt- or freshwater–but also in the terrestrial environment on rocks and soil, from desert to Antarctica. They may propagate in planktonic lifestyle or may live in microbial mats or biofilms. Their ubiquitous presence in the biosphere is based on various survival strategies, including the production of over 1,100 secondary metabolites (Dittmann et al., 2015), enabling them to compete for their ecological niches or to survive under difficult environmental conditions. Chemically mediated interactions, either positive or negative, between plants, plants and microbes, or microbe–microbe are termed as allelopathic (Rice, 1984; Weir et al., 2004). Among others, allelopathy is a strategy to fight competitors and predators (Leflaive and Ten-Hage, 2007). The ability of cyanobacteria to excrete allelopathic compounds, or allelochemicals, was discovered over 50 years ago in field-studies of freshwater cyanobacteria (Keating, 1977, 1978), but also described for marine cyanobacteria (Paz-Yepes et al., 2013). Allelochemicals from cyanobacteria, such as cyanobacterin from *Scytonema hofmanii* (Gleason and Paulson, 1984) and fischerellin A from *Fischerella muscicola* (Gross et al., 1991) often inhibit the photosystem II. The target organisms are other cyanobacteria, micro- and macro-algae as well as angiosperms (Leão et al., 2009). The majority of bioactive compounds, isolated mostly from few genera (*Lyngbya*, *Microcystis*, *Nostoc*, and *Hapalosiphon*), often have complex chemical structures and are mainly synthesized by gene clusters containing nonribosomal peptide synthetases (NRPS) and or polyketide synthases (PKS) (Dittmann et al., 2015). Although a variety of bioactive compounds have been isolated from cyanobacteria, only a few are considered to act as allelopathic inhibitors in the natural habitat of the producer strains [overview of allelopathic inhibitors from cyanobacteria by Leflaive and Ten-Hage (2007)]. Classification of a bioactive compound as an allelochemical can be controversial in certain cases, because it is difficult to simulate a complex ecosystem in the laboratory (Gross, 2003; Inderjit and Duke, 2003). The following aspects are important, when speculating about a possible role in allelopathic interactions: Allelopathic inhibitors must be excreted by the producer strain and not released by cell lysis, as it is the case for microcystins, which are regularly stored intracellularly (Schatz et al., 2007; Jüttner and Lüthi, 2008; Leão et al., 2009). Another aspect is the capability of the target organisms to take up the inhibitory compound, which is a less addressed topic (Inderjit and Duke, 2003). In the context of allelopathic interactions between cyanobacteria mediated by polar/hydrophilic compounds, the structure of their envelope plays an important role. Although cyanobacteria are regarded as Gram-negative bacteria, possessing an outer membrane, they also possess a thick peptidoglycan layer with a degree of crosslinking that resembles that from Gram-positive bacteria (Hansel and Tadros, 1998; Hoiczyk and Hansel, 2000; Castenholz, 2015). Molecules can pass the outer membrane via relatively unspecific porins, whereas transport over the cytoplasmic membrane is carried out via specific transporter, for example the fructose ABC-transporters (ATP-binding cassette transporters) (Ungerer et al., 2008) or transporters belonging to the major facilitator superfamily like the glucose permease Gtr (Schmetterer, 1990). The allelochemical microcin C possesses a peptide moiety, which is required for the uptake via an ABC-transporter into sensitive cells. Intracellularly, the peptide moiety is cleaved and converted to the mature inhibitor (Metlitskaya et al., 2006; Novikova et al., 2007).

**FIGURE 1:**
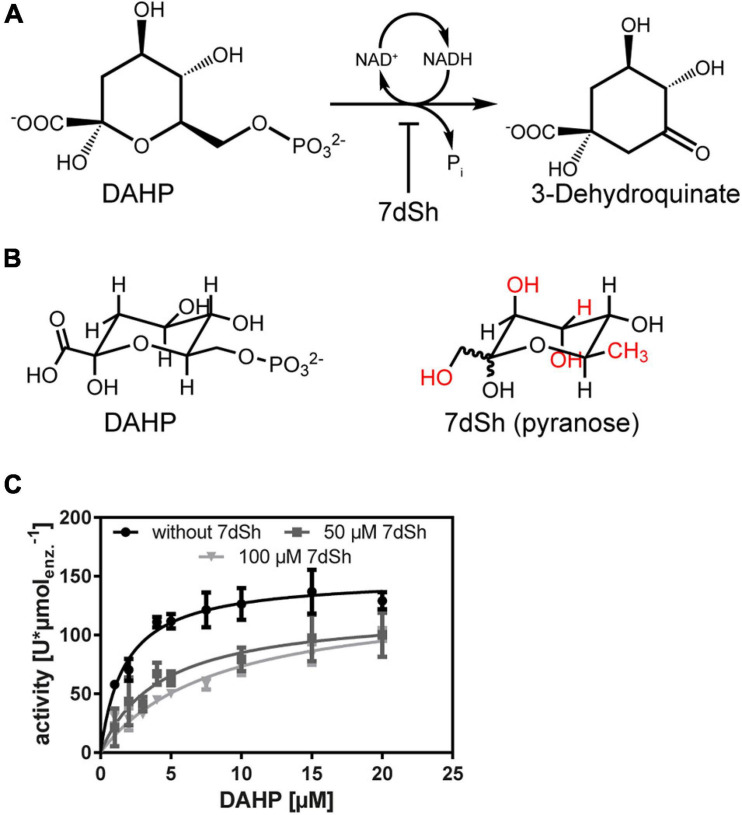
The NAD^+^-dependent 3-dehydroquinate synthase (DHQS) mediates the conversion of 3-deoxy-D-*arabino*-heptulosonate 7-phosphate (DAHP) into 3-dehydroquinate by the release of phosphate. 7dSh is an inhibitor of the DHQS-mediated reaction. **(A)** Schematic representation of the DHQS mediated reaction. **(B)** Structural comparison of DAHP and 7-deoxysedoheptulose (7dSh) in its pyranose form. **(C)** Michaelis-Menten of the *Av*DHQS-mediated conversion of DAHP into 3-dehydroquinate in the absence of 7dSh (black dots) or in the presence of 7dSh (dark grey squares–50 μM 7dSh, light grey triangles–100 μM 7dSh). Kinetic parameters are depicted in Table 3. Values represent mean and standard deviation of three independent replicates.

The common unicellular model organism *Synechococcus elongatus* PCC 7942 (thereafter *S. elongatus*) has a small, streamlined genome, which has no known gene cluster for the production of secondary metabolites (Shih et al., 2013; Copeland et al., 2014). Nevertheless, we have recently isolated and characterized a bioactive compound from the supernatant of this strain, which inhibits not only the growth of other cyanobacteria, but also the growth of fungi and plants (Brilisauer et al., 2019). The compound was characterized as a rare deoxy sugar–namely 7-deoxy-D-*altro*-2-heptulose (7-deoxysedoheptulose, 7dSh) (Brilisauer et al., 2019). Recently, we showed that 7dSh derives from 5-deoxyadenosine (5dAdo) (Rapp et al., 2021), an inhibitory by-product of radical SAM enzymes, which are involved in a multitude of reactions (Sofia et al., 2001), for example in the synthesis of the essential cofactors biotin and thiamine (Choi-Rhee and Cronan, 2005; Challand et al., 2010). 5dAdo is metabolized into 5-deoxyribose (5dR) and subsequently converted to 7dSh, by the sole activity of promiscuous enzymes, without a specific gene cluster being involved (Rapp et al., 2021). Therefore, 7dSh is not a “classical” secondary metabolite as it derives from a toxic waste product of the primary metabolism. 5dAdo is only converted to 7dSh under certain environmental conditions, suggesting an unknown regulatory mechanism for the formation of the “secondary” metabolite 7dSh (Rapp et al., 2021). 7dSh is most likely an antimetabolite of the shikimate pathway, as 7dSh treated cells strongly accumulate the structurally similar molecule 3-deoxy-D-*arabino*-heptulosonate 7-phosphate (DAHP), the substrate of the 3-dehydroquinate synthase (DHQS) (see Figure 1; Brilisauer et al., 2019). This NAD^+^-dependent enzyme catalyzes the conversion of DAHP into 3-dehydroquinate by the release of phosphate (Figure 1A). The shikimate pathway consists of seven enzymatic steps that convert erythrose 4-phosphate and phosphoenolpyruvate into chorismate, a precursor molecule for the synthesis of aromatic amino acids, folate cofactors and isoprenoid quinones. Additionally, 7dSh treated *Anabaena variabilis* cells had a significantly reduced content of aromatic amino acids (Brilisauer et al., 2019), further indicating that 7dSh is an inhibitor of this pathway. The pathway is only present in bacteria, including cyanobacteria, fungi, plants, and apicomplexans (Bentley, 1990; Roberts et al., 1998), thereby being a useful target for antibiotics, fungicides, and herbicides.

**TABLE 1:** Cyanobacterial strains used in this study.

**Strain**
*Synechococcus elongatus* PCC 7942
*Synechococcus elongatus* PCC 7942-7dSh^R^
*Synechococcus elongatus* PCC 7942 *Synpcc7942*_*0116*::*spec*^R^
*Synechococcus* sp. PCC 6301
*Synechocystis* sp. PCC 6803 GT
*Synechocystis* sp. PCC 6803 GT-7dSh^R^
*Synechocystis* sp. PCC 6803 GT *sll0771*::*spec*^R^
*Synechococcus* sp. PCC 7002
*Synechococcus* sp. PCC 6312
*Pleurocapsa minor* SAG 4.99
*Stanieria cyanosphaera* SAG 33.87
*Phormidium molle* SAG 26.99
*Leptolyngbya boryana* PCC 6306
*Oscillatoria acuminata* PCC 6304
*Anabaena* sp. PCC 7120
*Anabaena* sp. PCC 7120 (pRL1049-*frtRABC*)
*Anabaena variabilis* ATCC 29413
*Anabaena variabilis* ATCC 29413-7dSh^R^
*Nodularia sphaerocarpa* SAG 50.79
*Nostoc muscorum* SAG 1453-12a
*Scytonema hofmanii* PCC 7110
*Chlorogloeopsis fritschii* PCC 6912
*Fischerella muscicola* PCC 7414
*Mastigocladus laminosus* SAG 4.84

**TABLE 2:** Oligonucleotides used in this study.

**Name**	**Sequence (5′ → 3′)**
1_Avar AroB	**GAGAGACATATG**ATGACTTCTGTAATTAATGTGAATCTA
2_Avar AroB rev	**GAGAGACTCGAG**CATCTGCTGTAAAACTTGCCGAA
3_frtR_fw	**ACGGTTTCCCTCTACCGGGATCC**CACAGACCGAAGT GGAAATG
4_frtC_rev	**CGCAAGAGGCCCTTTCGTCTTCAAGAATTC**TGTTCAC GCAACGAGAAACC
5_pRL1049_fw	CGATCCCGCGAAATTAATAC
6_Ava2170_rev	TAGTTGACTTGTAAAGTTTTGCGTACTGAG
7_Gtr_rev	TTTGGGCCTCACTGGGTATC
8_Gtr_fw	GCAACTTGCCATAGGCTAAC
9_0771_up_fw	**AGCTCGGTACCCGGGGATCCT**GGGAAAGGAATTGAT CGG
10_0771_up_rev	**CTGCGTTCGGTCAAGAGCT**GTAAAGCTGAAATTGA AGAAG
11_0771_down_fw	**GAGATCACCAAGGTAGTCGGCAAATAA**TTAATTTTTTAT TTTGAGGGG
12_0771_down_rev	**ACGCCAAGCTTGCATGCCTGCA**CCACCAAACTTTGCA GAG
13_0116_up_fw	**AGCTCGGTACCCGGGGATCCT**TCCGCACCACTTCCC GTTTG
14_0116_up_rev	**CTGCGTTCGGTCAAGAGCT**CCCCGCTTCCCCCGTGG
15_0116_down_fw	**GAGATCACCAAGGTAGTCGGCAAATAA**CTATCTAAAC AGCAAATTAAC
16_0116_down_rev	**ACGCCAAGCTTGCATGCCTGCA**GAGGTTCCATCAGC ATAC
17_0116_seq_fw	TGTGGGTCGTTCGATTCC
18_0116_seq_rev	ATCGGAAGCCAAGTTAGC
19_Spec_fw	GAGCTCTTGACCGAACGCAG
20_Spec_rev	TTATTTGCCGACTACCTTGGTGATCTC
21_2170_fw	ACATTCGTTGGCTGACTG
22_2170_rev	AGCAGGCGTTTCTCATTC
23_2171_fw	ACGGCTGCTGACACTGATAC
24_2171_rev	GATCGCTTCTATGGCTTCTG
25_2172_rev	GATGCTCCAGTTGCATAGTG
26_2172_fw	TTTCTCCACCTGCGACTG
27_2173_fw	ACGAGCATCCCAAATCAC
28_2173_rev	CTGCGGAGTCTGTCAATC

*Overlapping fragments for Gibson Assembly are labelled in bold. Underlined sections indicate restriction sites.*

**TABLE 3:** Kinetic parameters of the *Av*DHQS mediated conversion of DAHP into 3-dehydroquinate in the absence or presence of the inhibitor 7dSh.

**7dSh (μM)**	***k*_*M*_ (μM)**	*v*_max_ (U*μmol_enz._^–^**^1^)**
–	1.8 ± 0.3	149.3 ± 6.1
50	4.3 ± 1.1	121.2 ± 10.9
100	8.2 ± 1.5	133.9 ± 10.9

In this study we confirmed the intracellular target of 7dSh biochemically using purified protein and identified the competitive nature of the inhibitor. Furthermore, we screened the spectrum of sensitive cyanobacteria and elucidated the uptake of 7dSh by two different types of transporters. Effective uptake of 7dSh leading to an intracellular enrichment is essential for the inhibitory effect of 7dSh. The characterization of the target but also the uptake mode is interesting for the ecological function of the inhibitor, but also relevant for potential application of 7dSh as an herbicide.

## Materials and Methods

### Cultivation

**FIGURE 2:**
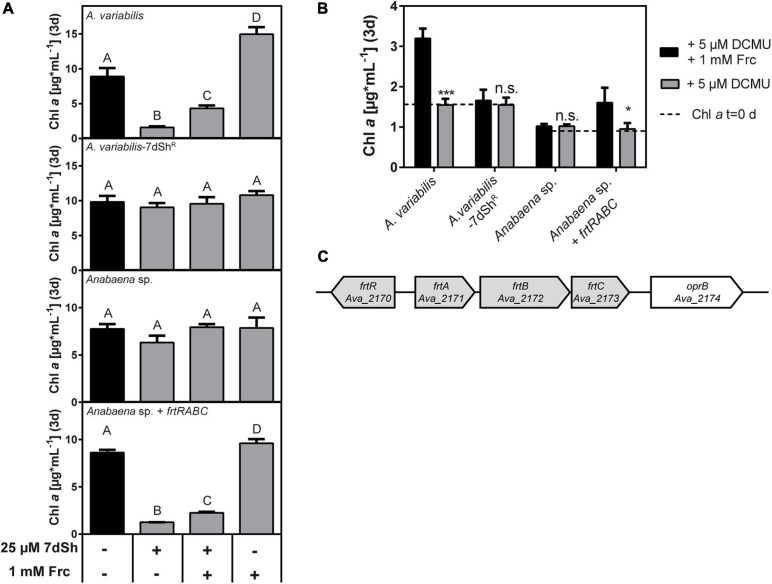
7-deoxysedoheptulose is taken up via the fructose ABC-transporter in *A. variabilis*. **(A)** Effect of 7dSh, 7dSh in combination with fructose and fructose alone on the growth of *A. variabilis*, *A. variabilis*-7dSh^R^, *Anabaena* sp. and *Anabaena* sp. expressing the fructose ABC transporter operon of *A. variabilis* (*Anabaena* sp. + *frtRABC*). The strains were inoculated with a chlorophyll *a* content of around 1 μg*mL**^–^**^1^ and cultivated for 3 days. Values shown in the graphs represent mean and standard deviation of three biological replicates. Statistical analysis was performed by using a one-way ANOVA followed by Tukey’s multiple comparison test. Means that were significantly different (*p*-value < 0.05) are marked with different capital letters. **(B)** Photoheterotrophic growth on fructose in the presence of the photosystem II inhibitor DCMU. The strains were inoculated with the indicated chlorophyll *a* content (dashed line) and cultivated for 3 days. Values shown in the graphs represent mean and standard deviation of three biological replicates. Significant photoheterotrophic growth was analyzed by comparing the growth in the presence of DCMU with or without fructose using an unpaired *t*-test (*p-value < 0.05; **p-value < 0.01; ***p-value < 0.001; n.s., not significant). **(C)** Genomic context of the fructose ABC-transporter *frtABC* operon including the regulator *frtR* in *A. variabilis*. The operon is clustered with the putative carbohydrate porin *oprB*.

Cyanobacterial strains (see Table 1) were cultivated under photoautotrophic conditions in BG11-medium (Rippka et al., 1979) supplemented with 5 mM NaHCO_3_, except for *Synechococcus* sp. PCC 7002 which was cultivated in a 1:1 mixture of BG11 and ASN III + vitamin B_12_ (10 μg^∗^mL^–1^) (Rippka et al., 1979). Cultivation was conducted at 27°C, constant illumination with light intensity of 30–50 μE (Lumilux de Lux, Daylight, Osram) and continuous shaking (125 rpm). Bioactivity assays were performed in a 24-well plate (except for *A. variabilis* and *Anabaena* sp. from Figure 2). The respective strains were inoculated with an optical density of OD_750_ = 0.05 in 1 mL BG11 and cultivated for 3–7 days at the conditions mentioned above. Stock solutions of 7dSh, fructose and glucose were set up in water and then applied to the cultures. Final concentrations are shown in the figures. 7dSh was synthesized as described in our previous publications (Brilisauer et al., 2019; Rapp et al., 2021). Cell density of the unicellular strains was determined by the measurement of the absorbance at 750 nm (Specord, Analytik Jena).

Bioactivity assays with *A. variabilis* and *Anabaena* sp. PCC 7120 were performed in shaking flasks with a cultivation volume of 5 mL (Figure 2). Flasks were inoculated with a chlorophyll *a* content of around 1.0–1.5 μg^∗^mL^–1^. Cell density of the filamentous cyanobacteria was determined by measuring the content of chlorophyll *a* as the determination of this is more precise in filamentous strains. In short: 1 mL of the culture was centrifuged (16.000×*g*, 5 min) and the supernatant was discarded. The pellet was extracted with 1 mL 90% (v/v) MeOH and incubated for 30 min in the dark. The cell debris was removed by centrifugation (16.000×*g*, 2 min). Chlorophyll *a* content was measured by the absorbance at 665 nm and the amount was calculated as described elsewhere (Mackinney, 1941).

For heterotrophic growth experiments, the cultures were incubated with fructose or glucose in the presence of the photosystem II-inhibitor DCMU [3-(3,4-dichlorophenyl)-1,1-dimethylurea] (Rippka, 1972). DCMU was dissolved in acetone and adjusted to a final concentration of 5 μM.

Spontaneous 7dSh-resistant mutants (7dSh^R^) of *A. variabilis*, *Synechocystis* sp. and *S. elongatus* were isolated from cultures that were cultivated at sublethal concentrations of 7dSh (10 μg^∗^mL^–1^ 7dSh for *A. variabilis* and 20 μg^∗^mL^–1^ for *Synechocystis* sp. and *S. elongatus*) and then plated on agar plates containing the same concentration of 7dSh. One single colony per strain was isolated and then used for bioactivity assays and sequencing (single genes: *gtr*/*sll0771* in *Synechocystis* sp., *frtRABC* in *A. variabilis*, and whole genome sequencing in *S. elongatus*).

### Cloning, Expression, Purification, and Activity of the DHQS From *A. variabilis*

The 3-dehydroquinate synthase (EC 4.2.3.4) of *A. variabilis* (Ava_4386) was amplified from genomic DNA with primers 1 and 2 (Table 2). Vector pET22b and the amplified gene was cut with NdeI/XhoI and ligated as described elsewhere and then transformed into *Escherichia coli* Top10. With vector pET22b, a His-Tag was introduced on the C-terminus of the protein. The vector was verified by sequencing (LightRun Tube, Eurofins Genomics, Ebersberg, Germany) and then transformed into *E. coli* BL21 (DE3). For protein expression the strain was cultivated in 400 mL LB at 37°C (150 rpm) until an optical density of around OD_600_ ≈ 0.8. Protein expression was induced with IPTG with a final concentration of 500 μM. After overnight cultivation at 20°C (150 rpm), the cells were harvested by centrifugation (6,000×*g*, 10 min, 4°C). The pellet was resuspended in 40 mL lysis buffer (50 mM *Tris*–HCl pH 7.5, 300 mM NaCl, 10 mM imidazole, protease inhibitor (complete ULTRA tablets, Roche) and DNAseI). Cell disruption was conducted via sonification (Branson, 5 mm tip). Cell debris was removed by centrifugation (40.000×*g*, 45 min, 4°C). The cell lysate was filtered with a syringe filter (0.45 μm) and applied to a HisTrap HP column (1 mL, GE Healthcare Life Science). After washing of the column, the protein was eluted with elution buffer (50 mM *Tris*–HCl, 300 mM NaCl, 250 mM imidazole, pH 7.5). Protein containing fractions were pooled and dialyzed overnight (ZelluTrans MWCO 3500 Da, Roth; in 50 mM *Tris*–HCl pH 8.0, 100 mM NaCl, 5 mM MgCl_2_, 1 mM DTT, 0.5 mM ETDA, 50% glycerol) and stored at –20°C. Purity was confirmed by SDS-PAGE. Protein concentration was determined with the Bradford method (RotiQuant, Roth).

In the 3-dehydroquinate synthase mediated reaction DAHP is converted into 3-dehydroquinate by the release of phosphate (see Figure 1A). The activity of DHQS was determined by phosphate release as described by Zhu et al. (2018) with malachite green (Phosphate Assay Kit, abcam, Cambridge, United Kingdom). The reaction was performed in a total volume of 200 μL in a 96-well plate. For this purpose, the prewarmed buffer (25 mM *Tris*–HCl pH 7.5 50 mM KCl) was mixed with 10 μM NAD^+^ (Roth), 1 mM DTT (Roth), 2 nM of the enzyme, and varying concentrations of DAHP (Carbosynth, Berkshire, United Kingdom). The reaction was conducted at 29°C. The reaction was stopped by the addition of 30 μL malachite green solution. After 30 min incubation in the dark at room temperature, phosphate release was measured by the absorbance at 650 nm in a microplate reader (Tecan Spark, Männedorf, Switzerland). For the determination of the kinetic parameters of the natural reaction, phosphate release was monitored after 5, 7, and 10 min of reaction. With the slope through this time points, *v*_max_ and *k*_*M*_ was calculated. Enzyme activities were therefore expressed as phosphate release in micromoles per minute per μmol of the enzyme (U^∗^μmol_enz._^–1^). The kinetic parameters were determined with GraphPad prism by fitting the data into the following equation: $v=\frac{v_{\text{max}}\times \left[S\right]}{\left(k_{m}+\left[S\right]\right)}.$

The inhibition constant *k*_*i*_ was calculated by using the following model: $k_{M\,\text{Obs}}=k_{M}\times \left(\frac{1+\left[I\right]}{k_{i}}\right)$ and $Y=\frac{v_{\text{max}}\times X}{\left(k_{M\,\text{Obs}}+X\right)}$, where *k*_*MObs*_ is the Michalis-Menten constant in the presence of a competitive inhibitor.

### (Partial) Sequencing of Spontaneous 7dSh-Resistant Mutants

To analyze the genomic background of the spontaneous *A. variabilis*-7dSh^R^ mutant, genomic DNA of the mutant and the wildtype was isolated and the genomic environment of the fructose ABC-transporter operon (*Ava_2170*-*Ava_2173*) was investigated (see Supplementary Figure 2). Gene specific primers were designed for each single gene of the operon, but also for the whole operon. A PCR with a Taq polymerase (RedTaq Mastermix, Genaxxon bioscience, Ulm, Germany) was performed to amplify these regions. The products were analyzed on an agarose gel.

Genomic DNA of the *Synechocystis* sp.-7dSh^R^ mutant and wildtype was amplified with a Q5-Polymerase (New England Biolabs, Ipswich, MA, United States) using primers 7+8 (sequence in Table 2). The product was analyzed by Sanger sequencing (LightRun Tube, Eurofins).

To obtain high quality genomic DNA of *S. elongatus* wildtype and *S. elongatus*-7dSh^R^, the extraction was performed with the DNeasy PowerLyzer Microbial Kit (Qiagen). Whole genome sequencing was performed by CD Genomics (“Microbial Whole Genome Sequencing”, New York, NY, United States).

### Construction of *Anabaena* sp. (pRL1049-*frtRABC*)

The fructose transporter operon from *A. variabilis* (*Ava_2170*-*Ava_2173*) was amplified from genomic DNA with the Gibson primers 3+4 (sequence in Table 2), adding overlapping fragments to the operon. Vector pRL1049, which is self-replicating in *Anabaena* sp. (Black and Wolk, 1994) was digested with EcoRI and BamHI. After that, the fragments were fused using Gibson Assembly (Gibson, 2011) and transformed in *E. coli* TOP10. The construct was verified by sequencing (LightRun Tube, Eurofins Genomics). Transformation in *Anabaena* sp. was performed by conjugation as previously described (Elhai and Wolk, 1988). The presence of the replicative plasmid in *Anabaena* sp. PCC 7120 was verified by a colony PCR using the primers 5+6 (sequence in Table 2). The strain was cultivated in the presence of 5 μg^∗^mL^–1^ spectinomycin and 5 μg^∗^mL^–1^ streptomycin.

### Construction of *Synechocystis* sp. *sll0771*::*spec*^R^ and *S. elongatus Synpcc7942_0116*::*spec*^R^

For the construction of the insertion mutants *Synechocystis* sp. *sll0771*::*spec*^R^ and *S. elongatus Synpcc7942_0116*::*spec*^R^ the respective gene was replaced with a spectinomycin resistance cassette. For this, flanking regions of both sides of the respective gene, 300–400 bp in length, were amplified from genomic DNA of the respective strain with primers adding overlapping fragments to the backbone vector or the spectinomycin resistance cassette. Primer 9+10 (for *Synechocystis* sp.) and 13+14 (for *S. elongatus*) were used to amplify the upstream fragments, primer 11+12 (for *Synechocystis* sp.) and 15+16 (for *S. elongatus*) for the downstream fragments (primer sequence see Table 2). The spectinomycin resistance cassette was amplified with the primer 19+20. For the replacement of the respective gene, the vector pUC19 (non-replicative in cyanobacteria) was digested with XbaI and PstI. All fragments (digested vector, upstream fragment, spectinomycin cassette, and downstream fragment) were fused using Gibson assembly (Gibson, 2011) and transformed into *E. coli* TOP 10. The plasmid was verified by Sanger sequencing (LightRun Tube, Eurofins Genomics). The plasmid was transformed into *Synechocystis* sp. or *S. elongatus* by using the natural competence of this strains. The exact procedure is described in our previous publication (Rapp et al., 2021). Integration of the plasmid and segregation was confirmed by colony PCR (9+12 for *Synechocystis* sp., 17+18 for *S. elongatus*, see Table 2). Both strains were cultivated in the presence of 20 μg^∗^mL^–1^ spectinomycin.

## Results

### 7dSh Is a Competitive Inhibitor of the 3-Dehydroquinate Synthase

*Anabaena variabilis* ATCC 29413 (thereafter *A. variabilis*) cultures treated with 7dSh strongly accumulate 3-deoxy-D-*arabino*-heptulosonate 7-phosphate (DAHP), which is the substrate of the 3-dehydroquinate synthase, the second enzyme in the shikimate pathway (Brilisauer et al., 2019; Figure 1A). Additionally, the content of aromatic amino acids is strongly decreased in 7dSh treated cells, whereas the pools of the non-aromatic amino acids are significantly elevated (Brilisauer et al., 2019). DAHP shows a similar structure as 7dSh in its pyranose form (Figure 1B, differences are labelled in red), suggesting that 7dSh is a structural analogue/antimetabolite of DAHP. To confirm the intracellular target and the inhibitory mode of 7dSh, an *in vitro* enzymatic inhibition assay was performed. Therefore, we cloned the DHQS gene from *A. variabilis* (*Ava_4386*, *Av*DHQS) into an *E. coli* overexpression vector, expressed it in *E. coli* BL21 (DE3) and purified the enzyme via its His-tag. Purity was confirmed by SDS-PAGE, where a band corresponding to the expected molecular weight of ∼39 kDa was observed (Supplementary Figure 1). In the DHQS mediated reaction, DAHP is converted into 3-dehydroquinate by the release of phosphate, which can be measured by means of the malachite green assay (Figure 1A). First, the kinetic parameters of the standard reaction were measured (Figure 1C, black dots; Table 3). For DAHP we obtained a *k*_*M*_-value of 1.8 ± 0.3 μM and a *v*_max_-value of 149.3 ± 6.1 U^∗^μmol_enz._^–1^. Following the addition of 50 or 100 μM 7dSh to the standard reaction, the *k*_*M*_-value drastically increased although *v*_max_ remained nearly constant (see Table 3 and Figure 1C, dark grey squares and light grey triangle). This confirmed the competitive nature of the DHQS inhibitor 7dSh and clearly showed that 7dSh, presumably in its furanose form, is a structural analogue of DAHP. From this data we calculated a *k*_*i*_ of 17.6 μM for 7dSh towards the DHQS from *A. variabilis*. Additionally, an IC_50_-value of 21.3 μM was determined (data not shown).

### Sensitivity of Different Cyanobacterial Strains Towards 7dSh Treatment

**FIGURE 3:**
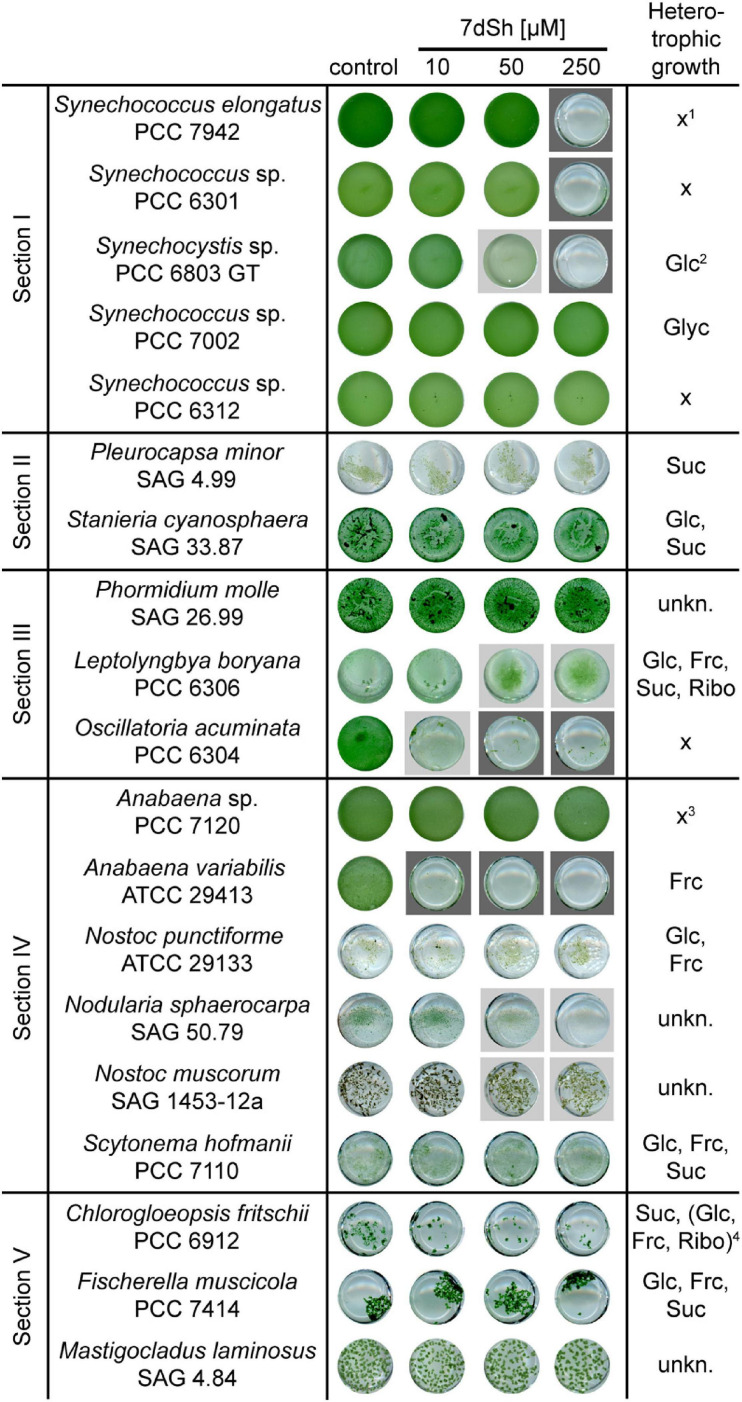
Effect of 7dSh toward selected cyanobacteria from all subsections. The respective strains were cultivated in a 24-well plate in BG11-medium in the presence of different 7dSh concentrations (0, 10, 50, and 250 μM). The cultures were inoculated at an optical density of OD_750_ = 0.05 and cultivated for 3–7 days. Dark grey background indicates a strong impact of 7dSh on the growth, whereas light grey labelling indicates a slight growth inhibition. The general capability of the strains for heterotrophic growth is depicted in the last column (Rippka et al., 1979). Abbreviations: Glc, glucose; Frc, fructose; Suc, sucrose; Ribo, ribose; unkn., unknown; *x*, no capability of heterotrophic growth. ^1^*S. elongatus* is capable of mixotrophic growth on fructose, sucrose, and xylulose (Peschek and Broda, 1973; McEwen et al., 2013). ^2^*Synechocystis* sp. is capable of fructose uptake, although fructose is toxic for this strain (Flores and Schmetterer, 1986). ^3^At very high fructose concentrations (50–200 mM) this strain is able to grow heterotrophically (Stebegg et al., 2012) or mixotrophically (Nieves-Morión and Flores, 2018). ^4^Only slow growth in the presence of the sugars in the brackets.

To elucidate the allelopathic potential of 7dSh, we examined the effect of the inhibitor on various cyanobacterial species belonging to all subsections of the phylum (Rippka et al., 1979). Therefore, we inoculated the strains in a 24-well plate in the presence of different 7dSh concentrations (0, 10, 50, and 250 μM) and cultivated them in continuous light until the control showed proper growth (Figure 3). Two of the strains, *A. variabilis* and *Oscillatoria acuminata* PCC 6304 were almost completely lysed by 10 μM 7dSh. *Synechocystis* sp. PCC 6803 GT (thereafter *Synechocystis* sp.), *Leptolyngbya boryana* PCC 6306 and *Nodularia sphaerocarpa* SAG 50.79 were moderately inhibited by 50 μM 7dSh. Growth inhibition in the 7dSh producer strains *S. elongatus* and *Synechococcus* sp. PCC 6301 was observed only at high 7dSh concentrations (250 μM). *Nostoc muscorum* SAG 1453-12a was slightly affected by showing a more yellowish phenotype at concentrations above 50 μM. Other cyanobacteria, especially those growing in macroscopic filaments, for example *Chlorogloeopsis fritschii* PCC 6912 and *Fischerella muscicola* PCC 7414, but also *Anabaena* sp. PCC 7120 (thereafter *Anabaena* sp.) and some unicellular cyanobacteria like *Synechococcus* sp. PCC 7002, *Synechococcus* sp. PCC 6312 and the two strains of subsection II (*Pleurocapsa minor* SAG 4.99, *Staniera cyanosphaera* SAG 33.87) were not affected by 7dSh even at high concentrations.

Taken together, these data indicate that the sensitivity to 7dSh may greatly vary from a cyanobacterial species to another. As some cyanobacteria are capable of heterotrophic growth (Rippka et al., 1979) and thereby endowed with the ability of sugar uptake (Schmetterer, 1990; Ungerer et al., 2008; Figure 3), we hypothesized that 7dSh sensitivity might be correlated with this ability, at least partially. Hence, we set out to test this hypothesis.

### Uptake of 7dSh in *A. variabilis* via the Fructose ABC-Transporter

To find a reason for the different sensitivities, we first focused on the highly sensitive *A. variabilis* and the close relative, but 7dSh insensitive *Anabaena* sp. strain (Figure 3). While the overall similarity of the two strains in homologous genes is 95% (Ungerer et al., 2008), their sequence similarity in the target of 7dSh, the DHQS enzyme, is around 99% (only four amino acids of the 363 are different). *A. variabilis* is known for its ability to use fructose as an additional carbon source or even grow heterotrophically on fructose (Haury and Spiller, 1981) as the strain contains an operon for a fructose ABC-transporter (Ungerer et al., 2008). *Anabaena* sp. has no homologous operon and is not able of heterotrophic growth at reasonable fructose concentrations (Rippka et al., 1979; Ungerer et al., 2008). Therefore, we assumed that this sugar transporter might play a role in the uptake of 7dSh. To verify this assumption, we performed bioactivity assays in the presence of 7dSh, fructose and a combination of both. After 3 days, the cell density (expressed as chlorophyll *a* content) was determined (Figures 2A,B). *A. variabilis* cells treated with 25 μM 7dSh showed a significantly reduced cell density, whereas the cell density of *Anabaena* sp. was not affected. The addition of a 40-fold higher concentration of fructose to *A. variabilis* alleviated the inhibitory effect of 7dSh to a certain extent but the culture did not regain the cell density of untreated cells. Additionally, we isolated a spontaneous 7dSh resistant *A. variabilis* mutant by cultivating an *A. variabilis* culture at sublethal concentrations of 7dSh (hereafter termed *A. variabilis*-7dSh^R^). The cell density of this mutant was not affected by the addition of 25 μM 7dSh. Furthermore, unlike the wildtype, the mutant was no longer capable of using fructose as an additional carbon source (Figure 2A) or of photoheterotrophic growth in the presence of the PS II inhibitor DCMU [3-(3,4-dichlorophenyl)-1,1-dimethylurea] (Figure 2B). To finally prove that the fructose ABC-transporter operon *frtRABC* is responsible for 7dSh uptake, we cloned this operon into a replicative plasmid (pRL1049) and introduced it into *Anabaena* sp. (thereafter named *Anabaena* sp. + *frtRABC*). The resulting strain gained sensitivity towards 7dSh, which could be alleviated by the addition of fructose as also shown for *A. variabilis* wildtype. Furthermore, this strain also gained the ability to use fructose as an additional carbon source (Figure 2A) or to grow photoheterotrophically on fructose in the presence of DCMU (Figure 2B), as also described in the literature (Ungerer et al., 2008). Additionally, we analyzed the genomic context of the *frtRABC* operon in *A. variabilis* wildtype and in *A. variabilis*-7dSh^R^. Therefore, we designed gene specific primers and amplified parts of the respective genes, but also the whole operon and analyzed the PCR fragments via agarose gel electrophoresis (see Supplementary Figure 2). The wildtype clearly showed the anticipated fragments for all the amplified genes (*Ava*_*2170*, *Ava*_*2171*, *Ava*_*2172*, and *Ava*_*2173*) as well as the fragment of the whole operon (*Ava*_*2170*-*Ava*_*2173*), whereas the mutant did not show any of the fragments, indicating a deletion of the whole operon in *A. variabilis*-7dSh^R^ (Supplementary Figure 2).

### Uptake of 7dSh in *Synechocystis* sp. via the Glucose Permease Gtr

**FIGURE 4:**
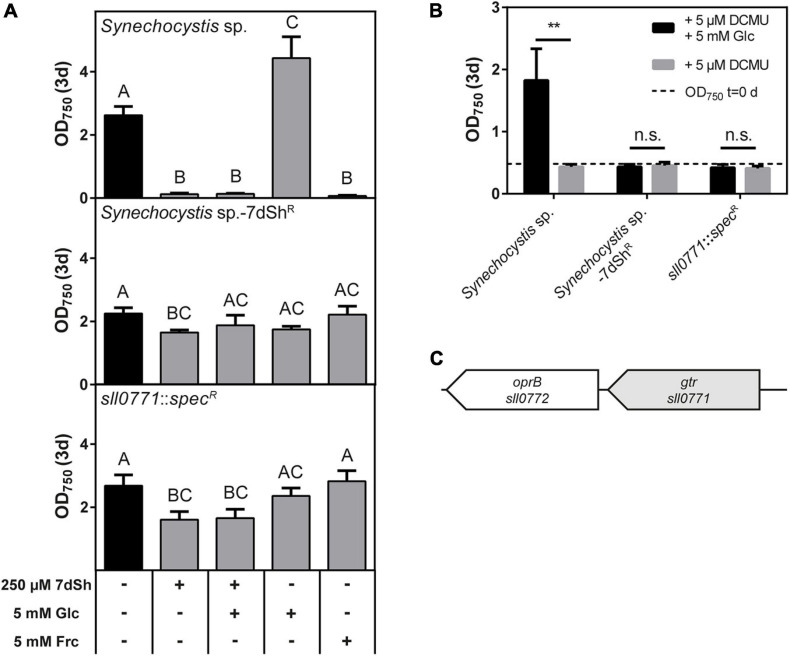
7-deoxysedoheptulose is taken up via the glucose permease Gtr in *Synechocystis* sp. **(A)** Effect of 7dSh, 7dSh in combination with glucose, glucose or fructose on the growth of wildtype *Synechocystis* sp., the 7dSh resistant mutant *Synechocystis* sp.-7dSh^R^ and the *gtr* insertion mutant *Synechocystis* sp. *sll0771*::*spec*^R^. The strains were inoculated at an optical density of OD_750_ = 0.05 and cultivated for 3 days. Values shown in the graphs represent mean and standard deviation of three biological replicates. Statistical analysis was performed by using a one-way ANOVA followed by Tukey’s multiple comparison test. Means that were significantly different (*p*-value < 0.05) are marked with different capital letters. **(B)** Heterotrophic growth on glucose by using the photosystem II inhibitor DCMU. The strains were inoculated at an optical density of 0.5 and cultivated for 3 days. Values shown in the graphs represent mean and standard deviation of three biological replicates. Significant heterotrophic growth was analyzed by comparing the growth in the presence of DCMU with or without fructose using an unpaired *t*-test (*p-value < 0.05; **p-value < 0.01; *** *p*-value < 0.001; n.s., not significant). **(C)** Genomic context of the glucose permease in *Synechocystis* sp. A putative carbohydrate porin is located downstream of *gtr*.

The unicellular cyanobacterium *Synechocystis* sp., a frequently used model strain, also showed sensitivity towards 7dSh treatment (Figure 3). The glucose-tolerant lab-strain can use glucose as an additional carbon source and can even grow heterotrophically on glucose (Rippka et al., 1979; Anderson and McIntosh, 1991). On the contrary, fructose is toxic for this strain (Flores and Schmetterer, 1986). Both, glucose and fructose are taken up by the glucose permease Gtr (also called GlcP or Sll0771), which belongs to the major facilitator superfamily (Joset et al., 1988; Zhang et al., 1989; Schmetterer, 1990). With this knowledge, we performed bioactivity assays with *Synechocystis* sp. in the presence of 7dSh, glucose, the combination of them and fructose and determined the cell density after 3 days (Figure 4). The addition of 250 μM 7dSh led to a significantly reduced cell density. Even the addition of the 20-fold amount of glucose did not alleviate the inhibitory effect of 7dSh. Additionally, we isolated a spontaneous 7dSh-resistant mutant by cultivating *Synechocystis* sp. at sublethal concentrations of 7dSh (hereafter termed *Synechocystis* sp.-7dSh^R^). The growth of this mutant was only slightly affected by the addition of 7dSh, but the mutant was no longer capable of using glucose as an additional carbon source or of photoheterotrophic growth (Figure 4A) on glucose in the presence of DCMU (Figure 4B). The wildtype showed a significantly enhanced optical density in the presence of glucose and during photoheterotrophic growth in the presence of glucose and DCMU. Furthermore, the *Synechocystis* sp.-7dSh^R^ was not inhibited by the addition of fructose, which showed toxicity in the wildtype. These findings suggest that the glucose permease is responsible for the uptake of 7dSh in *Synechocystis* sp. To confirm this assumption, we amplified and sequenced the gene of the glucose permease (*sll0771*) of wildtype and resistant mutant. It turned out that the mutant had acquired a single point mutation on position 1,174, containing a transition of thymine to cytosine. This results in an amino acid exchange from tryptophane to arginine located in a membrane-spanning helix at amino acid position 392, and therefore, of functional importance (Schmetterer, 1990). To confirm that the glucose permease Gtr is responsible for 7dSh uptake, the respective gene *sll0771* was replaced by a spectinomycin resistance cassette resulting in strain *Synechocystis* sp. *sll0771*::*spec*^R^. The growth of the mutant was only slightly affected by the addition of 7dSh, and the strain was no longer capable of using glucose as an additional carbon source or of heterotrophic growth on glucose (Figure 4). These findings clearly indicate that 7dSh is mainly imported via the glucose permease Gtr, but there might be also other transporters with lower affinity that can import 7dSh.

### 7dSh-Sensitivity of the Producer Strain *S. elongatus*

**FIGURE 5:**
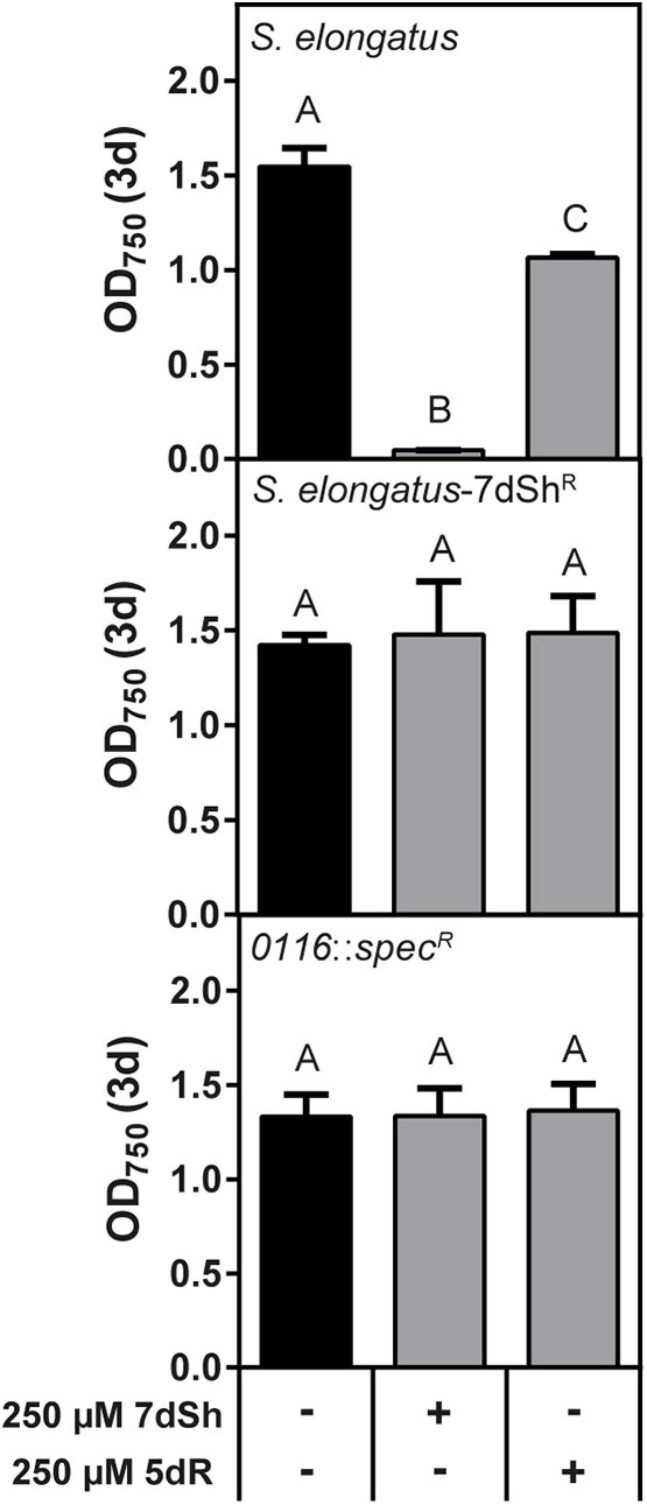
The putative ribokinase Synpcc7942_0116 is responsible for 5dR and 7dSh sensitivity in *S. elongatus*. Effect of 7dSh and 5dR on the growth on wildtype *S. elongatus*, the 7dSh resistant mutant *S. elongatus*-7dSh^R^ and the putative ribokinase insertion mutant *S. elongatus Synpcc7942_0116*::*spec*^R^. The strains were inoculated at an optical density of OD_750_ = 0.05 and cultivated for 3 days. Values shown in the graphs represent mean and standard deviation of three biological replicates. Statistical analysis was performed by using a one-way ANOVA followed by Tukey’s multiple comparison test. Means that were significantly different (*p*-value < 0.05) are marked with different capital letters.

*Synechococcus elongatus* is a natural producer of 7dSh and its precursor molecule 5-deoxyribose (5dR) (Brilisauer et al., 2019; Rapp et al., 2021). In our previous work we showed that both compounds are immediately excreted after formation, as none of these compounds accumulates intracellularly (Rapp et al., 2021). We furthermore showed that at the same time as 5dR is excreted, it is also imported and that both substances showed an inhibitory effect towards the producer at high concentrations (Rapp et al., 2021), indicating that there is also a mechanism for the uptake of both substances. By cultivating *S. elongatus* at sublethal 7dSh concentrations we isolated a spontaneous resistant mutant (*S. elongatus*-7dSh^R^), which was no longer sensitive towards 250 μM 7dSh, but also not sensitive towards 250 μM 5dR (Figure 5B). This suggests a common mechanism for the inhibitory effect of 5dR and 7dSh in *S. elongatus*. In contrast to the other strains analyzed above, *S. elongatus* is not able to grow heterotrophically (Rippka et al., 1979) and no sugar transporter is identified so far, although various ABC-type transporters and permeases are annotated in the genome of this strain. To elucidate the mechanism for 7dSh insensitivity, we performed a whole genome sequencing of the *S. elongatus*-7dSh^R^ and the wildtype. Compared to the annotated genome (GenBank accession No.: CP000100.1), our laboratory wildtype strain possesses three point mutations on the chromosome, leading to amino acid exchanges (see Supporting information, Table 1). In *S. elongatus*-7dSh^R^ four point mutations were identified, three of which also present in the wildtype. The additional point mutation, an exchange of thymine to adenine (position 923), resulting in an amino acid exchange from isoleucine to proline, is located at amino acid position 308 in gene *Synpcc7942*_*0116*. The gene is annotated as a sugar or nucleosidase kinase or belonging to ribokinase family (COG annotation) and it contains the PFAM motive pfkB of family carbohydrate kinase (PF00294). In the KEGG database it is annotated as a fructokinase (EC: 2.7.1.4) (Kanehisa and Goto, 2000). To confirm the role of this gene in 5dR/7dSh sensitivity, we replaced the gene with a spectinomycin resistance (*S. elongatus Synpcc7942*_*0116*::*spec*^R^) and performed growth experiments in the presence of 5dR and 7dSh (Figure 5C). Interestingly, the strain was no longer affected by 5dR or 7dSh (Figure 5), indicating an essential role of this gene product in the sensitivity of this strain regarding these two compounds.

## Discussion

Only few bioactive compounds from cyanobacteria are regarded as allelopathic inhibitors (Legrand et al., 2003; Leão et al., 2009). Here, we confirmed the allelopathic potential of 7dSh, a bioactive compound, which is actively formed and excreted by the unicellular cyanobacterium *S. elongatus*. 7dSh derives from a toxic waste product of the primary metabolism by solely promiscuous enzyme activity (Brilisauer et al., 2019; Rapp et al., 2021). First, we biochemically verified that 7dSh is an antimetabolite of the DHQS-mediated reaction, by mimicking the natural substrate DAHP (Figure 1). This is to the best of our knowledge the first example of an allelochemical targeting the shikimate pathway. Although a variety of allelochemicals are targeting the photosystem II (Gross, 2003), the shikimate pathway is an attractive target as all niche competitors of cyanobacteria possess this pathway (cyanobacteria, plants, but also other bacteria). The DHQS from *A. variabilis* was inhibited by 7dSh in a competitive manner and exhibited an inhibition constant (*k*_*i*_) of 17.6 μM or an IC_50_-value of 23.3 μM. 7dSh is most likely only active in its pyranose form, as the structural similarity to DAHP is obvious in this conformation (Figure 1B), although the majority of it is present in the furanose form (Brilisauer et al., 2019). For this reason, we assume, that only a minor part of 7dSh, the part, which is present in the pyranose form, is involved in the inhibition of the DHQS. According to literature, the hydroxyl group at C_5_, which has the same configuration in 7dSh and in the natural substrate DAHP, is important for binding in the active side of the enzyme. In this first step the hydroxyl group at C_5_ is oxidized by enzyme bound NAD^+^ (Carpenter et al., 1998). This ketone-NADH intermediate is bound very tightly to the enzyme (Bender et al., 1989), before β-elimination of the phosphate group occurs, which is blocked by various inhibitors, presumably also by 7dSh. The activity of 7dSh is comparable with other synthetic oxacyclic DHQS inhibitors (f.e. phosphonate), which display *k*_*i*_-values also in the lower μM range (Bender et al., 1989). Furthermore, the kinetic parameters of *A. variabilis* DHQS were similar to other organisms, for example *Actinidia chinensis*, which has a *k*_*M*_-value for DAHP of 1.3 μM (Mittelstädt et al., 2013).

Not all cyanobacteria are affected by 7dSh treatment: while several species are only affected in high concentrations (Figure 3), others are completely growth inhibited or even lysed at low μM concentrations of 7dSh (*A. variabilis*, *Oscillatoria accuminata*, Figure 3; Brilisauer et al., 2019). In complex communities involving different species, it is common that some species remain sensitive towards allelopathic inhibitors, whereas others develop strategies to tolerate the compounds (Bubak et al., 2020). The isolation of spontaneous 7dSh-resistant mutants demonstrates that cyanobacteria can easily adapt to the inhibitor. However, this goes hand in hand with the loss of the ability to use sugars as a(n) (additional) carbon source (see *A. variabilis*-7dSh^R^ and *Synechocystis* sp.-7dSh^R^), thereby losing a feature which could favor growth under certain nutritional conditions or in plant symbiosis (Ekman et al., 2013). We hypothesize that *S. elongatus* might compensate its inability to grow heterotrophically by excreting 7dSh, which then inhibits other cyanobacteria thereby hindering their overgrowth when sugars are present. Interestingly, some strains capable of heterotrophic growth on sugars, thereby necessarily possessing sugar uptake systems, are not affected by 7dSh (e.g., *Staniera cyanosphera*), suggesting that these strains might have developed mechanisms to grow heterotrophically while avoiding 7dSh inhibition. Additionally, heterotrophic bacteria are at most weakly affected by 7dSh [e.g., *Gluconobacter oxydans* is not inhibited by 7dSh (Brilisauer et al., 2019)]. For *E. coli* a weak growth inhibition could be observed when cultivated in minimal medium, but not in complex medium (Supplementary Figure 3). It might be possible that some heterotrophic bacteria are able to metabolize 7dSh, as it is reported that the cocultivation of cyanobacteria with heterotrophic bacteria can support the growth of the cyanobacteria (Zheng et al., 2018; Gao et al., 2020).

With growth experiments in the presence of 7dSh and/or fructose (Figure 2), we identified the *frtRABC* operon, encoding for the ABC-type fructose transporter and a *lacI*-like regulatory gene *frtR* (Ungerer et al., 2008), as the transporter for 7dSh uptake in *A. variabilis*. The promiscuous uptake of 7dSh by this transporter is quite effective as strong DAHP accumulation could be observed within 1 h, whereas in control cultures no DAHP was detectable (Brilisauer et al., 2019). Additionally, fructose in a 40-fold higher concentration does only partly alleviate the toxic effect of 25 μM 7dSh, indicating an effective and rapid uptake of 7dSh in *A. variabilis* cells. With this, we assume that either 7dSh has a high affinity towards the fructose transporter [*k*_*M*_-value for fructose 140 μM (Haury and Spiller, 1981)] or 7dSh quickly increases the expression of the transporter as also reported for fructose (Ungerer et al., 2008). The effect of 7dSh, whether it shows bacteriostatic or bactericidal activity, is strongly dependent on the optical density of the cultures (Brilisauer et al., 2019), which clearly showed that the effect of 7dSh strongly depends on the amount of 7dSh per cell. This indicates that the intracellular concentration of 7dSh should be by orders of magnitude higher than the applied extracellular concentration and explains that extracellular concentrations of 7dSh, which were far below the *k*_*i*_-value, can have detrimental effects on *A. variabilis*. The effective and rapid uptake of 7dSh leading to a strong intracellular enrichment is thereby essential for the inhibitory activity of 7dSh. Although 7dSh is a competitive inhibitor, which should be displaced by increasing DAHP concentrations, we assume that the bactericidal effects of 7dSh are additionally caused by strong metabolic perturbations, which are also described for other compounds targeting the shikimate pathway. The physiological effects of glyphosate (targeting aromatic amino acid synthesis) or the acetolactate synthase (ALS) inhibitor Imazamox (targeting branched-chain amino acid synthesis) are broader than the sole depletion of amino acids, resulting for example in the use of less efficient metabolic pathways (Orcaray et al., 2012; Zulet et al., 2015). Additionally, we cannot exclude that 7dSh has also other, unknown side targets. 7dSh can sneak into cells of target organisms not only via the fructose ABC-transporter: it can also be imported by a structural and functional different sugar transporter–the well-characterized glucose permease of *Synechocystis* sp. (Flores and Schmetterer, 1986; Joset et al., 1988; Schmetterer, 1990). Besides its ability to transport glucose (*k*_*M*_ = 0.58 mM), also the glucose analogue 3-*O*-methyl-D-glucose (*k*_*M*_ = 1.66 mM) and fructose, which is even toxic for *Synechocystis* sp., can be taken up (Joset et al., 1988). It is obvious that *Synechocystis* sp. possesses also other transporters, which can take up 7dSh with a poorer efficiency as the growth of the *gtr* insertion mutant (*Synechocystis* sp. *sll0771::spec*^R^) is slightly impaired by 7dSh (Figure 4A). This is underlined by the fact that a *gtr*^–^ mutant gained the ability to grow heterotrophically on fructose, although for the wildtype fructose is toxic (Stebegg et al., 2019). Directly adjacent to the *frtRABC* operon in *A. variabilis* and adjacent to the glucose permease in *Synechocystis* sp., we noted the presence of a gene encoding for a putative carbohydrate porin (*Ava*_2174, *sll0772*) (Figures 2C, 4C) suggesting that porins might be responsible for the permeation via the outer membrane. *Sll0772* and *ava_2174* show high sequence similarity to a porin-encoding gene from *Nostoc punctiforme* ATCC 29133 (*N. punctiforme*), which is also clustered with a fructose ABC transporter and was shown to be responsible for permeation of the sugar through the outer membrane (Ekman et al., 2013). Interestingly, *N. punctiforme* is not affected by 7dSh treatment although it has a similar fructose ABC-transporter as *A. variabilis* and additionally a clustered glucose permease (Ekman et al., 2013). We hypothesize that this might be due to a different regulation, as the expression of the fructose transporter in *A. variabilis* is regulated by FrtR (Ungerer et al., 2008), whereas the homologue in *N. punctiforme* HrmR, does not appear to be involved in the regulation of the fructose transporter (Ekman et al., 2013). Besides lower uptake of 7dSh, there are also other possibilities which can result in insensitivity towards 7dSh, e.g., excretion systems, which might be especially present in strains with a large genome. This is underlined by the fact that also other complex, multicellular cyanobacteria (e.g., *Chlorogloeopsis fritschii* PCC 6912, *Fischerella muscicola* PCC 7414), which are able to use various sugars for heterotrophic growth are not affected by 7dSh treatment. We have to add that 7dSh might be not exclusively transported by sugar transporters, but also by other transporters. The uptake of 7dSh in the producer strain *S. elongatus* remains unclear, especially as the strain also possesses an effective, but unidentified excretion system, which leads to an immediate excretion of intracellularly formed 7dSh (Rapp et al., 2021). However, it seems that the strain possesses unidentified sugar transporters, which presumably allow the strain to grow mixotrophically on fructose, glucose, sucrose and xylose to a certain extent (Peschek and Broda, 1973; McEwen et al., 2013). The involvement of the putative ribokinase in 7dSh and 5dR-sensitivity should be further investigated. It is possible that in this strain phosphorylated 5dR and 7dSh, which cannot be excreted, act as inhibitors. For *A. variabilis* we exclude that phosphorylated 7dSh plays a role in the 7dSh-triggered inhibition. By having a look in previous HRLC-MS data, where we examined the metabolome of 7dSh treated and untreated *A. variabilis* cells, we clearly see an accumulation of a compound with a *m/z* ratio corresponding to the sum formula of 7dSh (C_7_H_14_O_6_ [M+H, M+Na]^+^) in 7dSh treated cells, but not in untreated control cultures. A compound with a *m/z* ratio of phosphorylated 7dSh (C_7_H_15_O_9_P [M+H, M+Na]^+^) neither accumulates in 7dSh treated nor in untreated cells (data not shown). We conclude that the extraction process does not destroy phosphorylated compounds, as we observe a strong accumulation of a phosphorylated compound with the sum formula C_7_H_13_O_10_P [M+H, M+Na]^+^, corresponding to the *m/z* ratio of DAHP in 7dSh treated cells. Additionally, during the elucidation of the biosynthesis of 7dSh, we were able to measure phosphorylated 5dR in crude extracts incubated with 5dAdo, with this method (Rapp et al., 2021).

The main problem of allelopathic interactions in aquatic ecosystems is the dilution of the allelochemical, thereby causing a reduction of the effective concentrations (Gross, 2003). Although *S. elongatus* was originally isolated from freshwater and is commonly cultivated in its planktonic lifestyle in the laboratory (Golden, 2019), various authors reported that *S. elongatus* can also exhibit a terrestrial lifestyle, colonizing soil, rocks, humid stonewalls, and even caves in microbial mats and biofilms (Schlösser, 1994; Czerwik-Marcinkowska and Mrozińska, 2011; Mattern and Mareš, 2018; Yang et al., 2018; Conradi et al., 2020). Although 7dSh is isolated from planktonic cells, we suggest that 7dSh as an allelopathic inhibitor might play a more important role in its terrestrial and community-/biofilm-forming lifestyle, where it accumulates in high concentrations. One of the two most sensitive strains in this study, *Oscillatoria accuminata*, also colonizes soil (strain information for SAG 1449-3 on the website). The soil bacterium *Streptomyces setonensis* produces significantly higher amounts of 7dSh (more than 10-fold compared to *S. elongatus*) (Rapp et al., 2021), suggesting that 7dSh excretion is a more common strategy in allelopathic interactions in a terrestrial lifestyle. It is possible that *S. elongatus* might be able to produce greater amounts of 7dSh under other conditions, because in our laboratory conditions only a minor part of 5dAdo is converted into 7dSh (Rapp et al., 2021). Besides the competition with other cyanobacteria, *S. elongatus* might also compete with angiosperms regarding light. 7dSh also strongly inhibits the growth of germinating *A. thaliana* seedlings, either on agar plates, but also in soil, where a significant growth reduction was observed already at 25 μM (Brilisauer et al., 2019), suggesting that 7dSh might also play a role in niche protection or competition for light with angiosperms. It is obvious that 7dSh is also taken up by (a) sugar transporter(s) in *A. thaliana* as the effect of 7dSh on germinating seedlings is strongly alleviated when sucrose is present (Supplementary Figure 4). 7dSh showed a similar effect on *A. thaliana* seedlings as the commercially available herbicide glyphosate (Brilisauer et al., 2019). As plants possess monosaccharide transporters, belonging to the major facilitator superfamily, for the distribution of sugars (Williams et al., 2000), we speculate that 7dSh might exhibit systemic toxicity which is essential for being an effective herbicide. The verification of the target of 7dSh, and the deeper understanding how 7dSh is taken up might also help to further develop 7dSh as an herbicide, as the uptake of an herbicide is a suitable target for the development of resistant crops. The exact uptake mechanism of glyphosate in plants is not yet known, but only recently a transporter for glyphosate uptake in *B. subtilis* was identified (Wicke et al., 2019).

With the identification of different sugar transporters responsible for 7dSh uptake and the fact that 7dSh is actively excreted by *S. elongatus*, we identified the allelopathic function of this rare deoxy-sugar. As it is also excreted by *S. setonensis* we suggest that formation and excretion of 7dSh is a more common mechanism in niche protection.

## Data Availability Statement

The original contributions presented in the study are included in the article/Supplementary Material, further inquiries can be directed to the corresponding author.

## Author Contributions

JR designed, performed, interpreted the experiments, and wrote the manuscript. BW and KB performed, interpreted, and discussed the experiments. KF supervised the study and supported the manuscript writing. All authors contributed to the article and approved the submitted version.

## Conflict of Interest

Eberhard Karls University of Tuebingen, KB, KF have filed a patent application that covers 7dSh, 7dSh analogs and their use (EKUT-0365, German patent application number DE10 2017 01 898.1, International patent application number PCT/EP2018/082440). The remaining authors declare that the research was conducted in the absence of any commercial or financial relationships that could be construed as a potential conflict of interest.
