# Supplementary material for: In vivo Inhibition of the 3-Dehydroquinate Synthase by 7-Deoxysedoheptulose Depends on Promiscuous Uptake by Sugar Transporters in Cyanobacteria
Source: Front Microbiol. 2021 Jun 23;12:692986. doi: 10.3389/fmicb.2021.692986 (PMC8261047; doi:10.3389/fmicb.2021.692986)
Supplement: Supplementary file 1 [file Data_Sheet_1.PDF]

Supporting information for:

In vivo inhibition of the 3-dehydroquinate synthase by 7-deoxysedoheptulose depends on promiscuous uptake by sugar transporters in cyanobacteria

Johanna Rapp, Berenike Wagner, Klaus Brilisauer, Karl Forchhammer

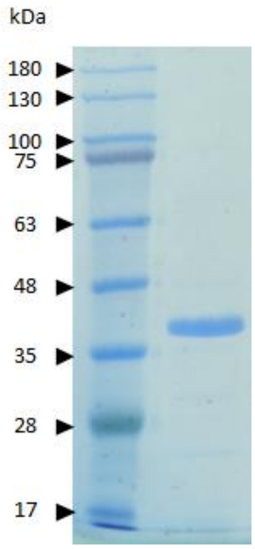

Figure S1: Coomassie-stained SDS-PAGE gel of purified *Av*DHQS. The protein was expressed in *E. coli* BL21 (DE3) with a C-terminal His-Tag and purified via Ni^{2+} affinity chromatography. ~0.5 µg of protein was applied to a 12 % SDS-PAGE gel.

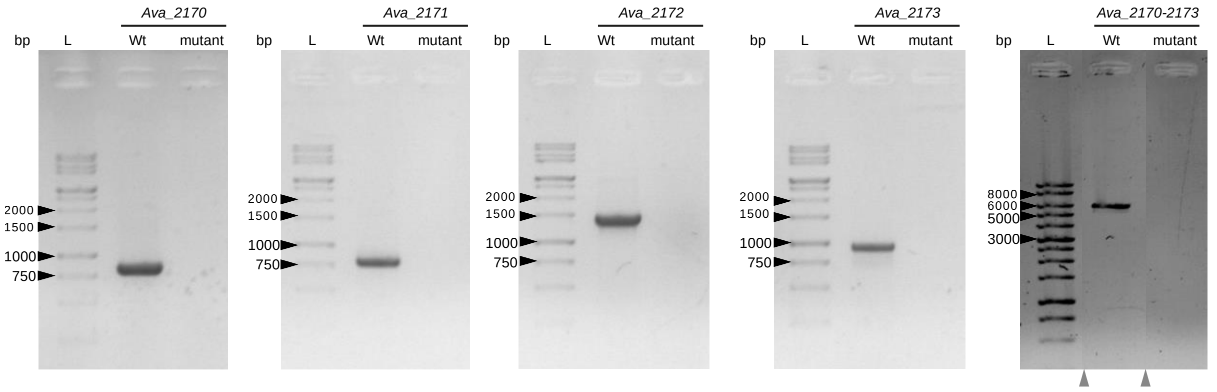

Figure S2: Analysis of PCR fragments of the *frtRABC* operon (*Ava_2170-2173*) from genomic DNA of *A. variabilis* (Wt) and a spontaneous 7dSh-resistant mutant (mutant) visualized by agarose gel electrophoresis. Gene specific primers were used in a PCR reaction with genomic DNA (50 ng DNA, Red Taq Mastermix, Genaxxon). *Ava_2170* – primer 21+22 (expected band size: 863 bp), *Ava_2171* – primer 23+24 (expected band size: 735 bp), *Ava_2172* – primer 25+26 (expected band size: 1510 bp), *Ava_2173* – primer 27+28 (expected band size: 946 bp), *Ava_2170*-*Ava_2173* – primer 3+4 (expected band size: 5574 bp). To analyse the presence of the single genes, primers lying inside the respective gene were used. For the amplification of the whole operon, primers lying 200 bp up- and downstream of the operon were used. Primer sequences are shown in Table 3. L - DNA ladder 1 kb (Genaxxon). Splice borders are labelled with grey triangle, but marker and samples were run on the same gel.

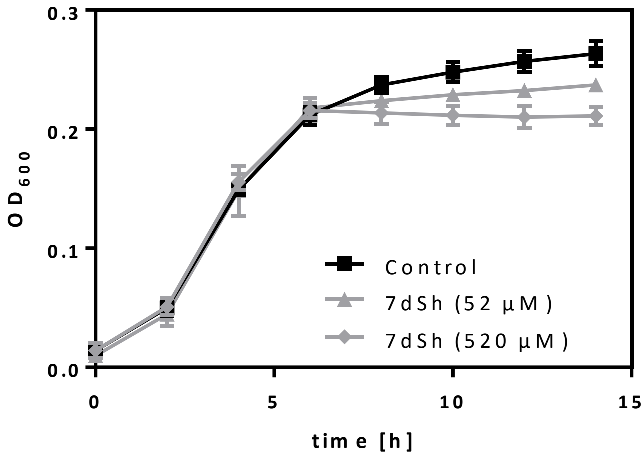

Figure S3: Growth of *E. coli* in minimal medium (M9) supplemented with 0.5 mg/mL fructose and 1 mg/mL casamino acids in the presence or absence of 7dSh. *E. coli* K12 was inoculated with an optical density of OD_{600}=0.02 in a 96-well plate and cultivated at 37 °C and constant shaking (120 rpm). Values represent mean and standard deviation of seven biological replicates. *E. coli* cells growing in complex media (LB) is not affected by the addition of 7dSh (data not shown).

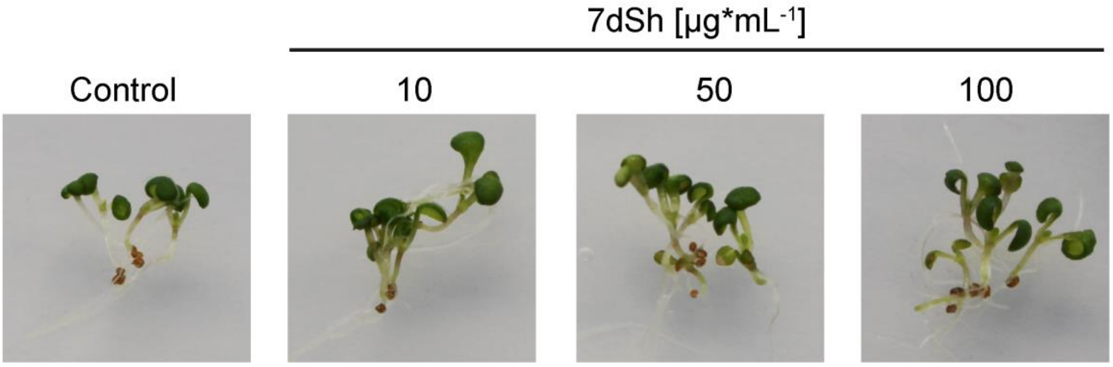

Figure S4: Effect of 7dSh on the germination of *A. thaliana* seedlings in liquid Murashige and Skoog Basal Medium containing 10 mg/mL sucrose after 7 days of cultivation in day/night cycle. In the absence of sucrose, the germination of *A. thaliana* seedlings in the presence of 7dSh is strongly decreased (Brilisauer et al., 2019).

Table 1: Results of the whole genome sequencing of *S. elongatus* and a spontaneous 7dSh-resistant mutant.

		Nucleotide in sense direction			Affected base triplet and corresponding amino acid in					
**Position on chromosome**	**Gene**	**reference genome**	**wildtype**	**resistant mutant**	**reference genome**		**wildtype**		**resistant mutant**	
92978	Synpcc7942_0095 compl.	T	**C**	**C**	C**A**G	Q (Gln)	C**G**G	R (Arg)	C**G**G	R (Arg)
115221	Synpcc7942_0116	T	T	**A**	A**T**C	I (Ile)	A**T**C	I (Ile)	A**A**C	N (Asn)
924962	Synpcc7942_0918	T	**C**	**C**	C**T**G	L (Leu)	C**C**G	P (Pro)	C**C**G	P (Pro)
2440364	Synpcc7942_2373 compl.	C	**T**	**T**	G**G**T	G (Gly)	G**A**T	D (Asp)	G**A**T	D (Asp)

REFERENCES

Brilisauer, K., Rapp, J., Rath, P., Schöllhorn, A., Bleul, L., Weiß, E., et al. (2019). Cyanobacterial antimetabolite 7-deoxy-sedoheptulose blocks the shikimate pathway to inhibit the growth of prototrophic organisms. *Nat. Commun.* 10, 545. doi: 10.1038/s41467-019-08476-8
